# Rosmagain™ as a Natural Therapeutic for Hair Regrowth and Scalp Health: A Double-Blind, Randomized, Three-Armed, Placebo-Controlled Clinical Trial

**DOI:** 10.7759/cureus.85906

**Published:** 2025-06-13

**Authors:** Maheshvari N Patel, Natasha Tuli, Nayan Patel, Apeksha Merja

**Affiliations:** 1 Clinical Research, NovoBliss Research, Ahmedabad, IND; 2 Pharmacology, Swaminarayan University, Ahmedabad, IND; 3 Research and Development, Soulflower Pvt Ltd, Mumbai, IND; 4 Clinical Research Operations, Novobliss Research Pvt Ltd, Ahmedabad, IND; 5 Clinical Research, Novobliss Research Pvt Ltd, Ahmedabad, IND

**Keywords:** alopecia, hair growth, hair length, hair strength, scalp condition

## Abstract

Introduction

Alopecia is a common condition marked by progressive hair loss, influenced by aging, genetics, hormones, and environmental factors. It affects individuals of all ages, often impacting self-esteem and quality of life. Rosemary and lavender oils have gained attention for their antioxidant, anti-inflammatory, and antimicrobial properties, promoting scalp health, follicular stimulation, and hair regrowth. While rosemary oil enhances circulation and extends the anagen phase, lavender oil soothes the scalp and supports hair growth. This study evaluates the efficacy and safety of rosemary-lavender oil and rosemary-castor oil in comparison of coconut oil in promoting hair growth and scalp health.

Method

This prospective, double-blind, randomized, three-arm, placebo-controlled clinical trial evaluated the efficacy and safety of rosemary-lavender oil and rosemary-castor oil, in comparison with coconut oil for hair growth over 90 days. This study was conducted per International Council for Harmonisation of Technical Requirements for Pharmaceuticals for Human Use (ICH) Good Clinical Practice (GCP), Declaration of Helsinki (2013), and Indian Council of Medical Research (ICMR) guidelines, the study received ethical approval (ACEAS IEC, February 10, 2024) and was registered with Clinical Trials Registry - India (CTRI; CTRI/2024/07/070860). Ninety healthy participants (18-55 years) were randomized equally. Study assessed the hair growth rate, thickness, density, length, scalp condition, and anagen:telogen (A:T) ratio using a phototrichogram (CASLite Nova, Catseye Systems & Solutions Pvt Ltd, Navi Mumbai, India). Visual/tactile assessments were done by a certified dermatologist. Statistical analysis used SPSS v29.0.1.0 (IBM Inc., Armonk, New York) and Excel 2019 (Microsoft, Redmond, Washington), applying paired t-tests (p< 0.05).

Result

The study demonstrated that rosemary-lavender oil and rosemary-castor oil significantly improved hair growth rate, thickness, density, length, and reduced hair fall compared to coconut oil (p<0.0001). Hair growth rate increased 0.22 ± 0.04 mm/day to 0.34 ± 0.05 mm/day or 57.73% (p<0.0001) change from baseline (CFB) in rosemary-lavender oil and 0.23 ± 0.04 mm/day to 0.33 ± 0.05 mm/day or 47.59% CFB (p<0.0001) in rosemary-castor oil. Hair thickness improved by 68.70% and 66.07% (p<0.0001), while hair density increased by 32.21% and 32.15%, respectively (p< 0.0001). Hair length showed a 28.78% and 37.67% increase, while hair fall reduction exceeded 40% in both rosemary groups (p<0.0001).

Conclusion

The findings demonstrate that rosemary-lavender, Rosmagain™, and rosemary-castor oils significantly provide beneficial effects in hair growth parameters. Their ability to enhance hair thickness, density, length, and reduced hair fall suggests they may serve as effective natural therapeutic alternatives for hair regrowth. With a favourable safety profile, these formulations offer a promising plant-based solution for good hair and scalp health. This formulation is designed to restore balance to the hair growth cycle, creating an optimal scalp environment for sustained and healthy hair growth.

## Introduction

Alopecia, commonly known as hair loss, is a condition characterized by partial or complete hair loss from areas where hair normally grows [1,2]. It is a natural and often progressive phenomenon influenced by various intrinsic and extrinsic factors. While hair loss can be a normal physiological occurrence, it may also indicate underlying pathological conditions [3]. Hair plays a significant role in personal identity, aesthetics, and social perception, making hair loss a common concern among individuals across different age groups [4,5].

Alopecia is broadly classified into two categories: non-scarring alopecia and scarring alopecia. Non-scarring alopecia includes conditions such as androgenetic alopecia, alopecia areata, telogen effluvium, and traction alopecia, where the hair follicles remain intact and capable of regrowth [6,7]. In contrast, scarring alopecia (cicatricial alopecia) is characterized by irreversible follicular destruction due to inflammatory processes, as seen in lichen planopilaris and discoid lupus erythematosus [8]. Additionally, physiological hair loss, often termed normal alopecia, occurs as part of the natural hair cycle and is influenced by aging, seasonal changes, and hormonal fluctuations.

The clinical presentation of alopecia varies depending on its type and underlying cause. Common signs include increased hair shedding, thinning hair, receding hairline, bald patches, and changes in hair texture [9]. In non-scarring alopecia, hair loss is often diffuse or patterned, while scarring alopecia may present with scalp erythema, scaling, or follicular atrophy. Alopecia, on the other hand, is typically characterized by daily hair shedding without significant scalp abnormalities or progressive hair thinning.

Several factors contribute to hair loss, including genetic predisposition, hormonal changes, nutritional deficiencies, psychological stress, medications, and environmental influences. Genetic factors play a crucial role in androgenetic alopecia, where individuals with a family history of hair loss are more susceptible [10]. Hormonal imbalances, particularly fluctuations in androgens, thyroid hormones, and estrogen, can disrupt the hair growth cycle [11]. Nutritional deficiencies, such as insufficient iron, biotin, or vitamin D intake, may exacerbate hair shedding [12]. Psychological stress is another contributing factor, often triggering telogen effluvium, a condition where a larger proportion of hair follicles prematurely enter the resting phase [13]. Certain medications, including chemotherapy, immunosuppressants, and hormonal treatments, are also known to induce hair loss [14]. Additionally, environmental and lifestyle factors such as pollution, excessive hairstyling, and chemical exposure can damage hair and accelerate shedding [15].

The pathophysiology of alopecia varies depending on its underlying cause. In androgenetic alopecia, dihydrotestosterone (DHT) binds to androgen receptors in susceptible hair follicles, leading to follicular miniaturization and a shortened anagen phase [16]. In autoimmune conditions like alopecia areata, T-cell-mediated inflammation targets hair follicles, resulting in non-scarring hair loss [17]. In normal alopecia, hair shedding occurs due to the natural transition of hair follicles through the hair cycle without any inflammatory or pathological processes.

Hair growth follows a cyclical pattern consisting of three main phases: anagen (growth phase), catagen (transition phase), and telogen (resting phase). The anagen phase is the longest and most active phase, lasting between two to seven years, during which new hair is produced. The catagen phase is a short transitional period of two to three weeks, where the hair follicle begins to regress. The telogen phase, lasting approximately three to four months, is the resting period, after which hair shedding occurs and new hair growth begins. On a daily basis, approximately 10-15% of scalp hair is in the telogen phase, leading to a normal shedding of 50-100 hairs per day. This process is essential for hair renewal and is not indicative of pathological hair loss [18,19].

Various natural remedies, including essential oils, have been studied for their potential role in promoting hair growth and reducing hair loss. Lavender oil, known for its antimicrobial and anti-inflammatory properties, has been found to enhance hair growth by increasing the number of hair follicles and deepening the anagen phase, as demonstrated in animal studies [20,21]. Rosemary oil has been shown to improve hair regrowth in androgenetic alopecia by promoting circulation in the scalp and inhibiting DHT, with studies suggesting its efficacy comparable to minoxidil [22]. Castor oil, rich in ricinoleic acid, has been traditionally used to nourish hair and enhance its density, though scientific evidence supporting its direct role in alopecia management remains limited [23]. These natural treatments are increasingly being explored as complementary therapies to conventional hair loss treatments.

Understanding normal alopecia and its distinction from pathological hair loss is crucial for accurate diagnosis and management. By recognizing the physiological aspects of hair shedding and its influencing factors, clinicians can guide patients in differentiating between normal hair loss and conditions requiring medical intervention. This manuscript aims to explore the characteristics of normal alopecia and its clinical relevance in dermatology.

Given the growing interest in natural alternatives for hair loss management, our clinical study evaluates the efficacy of rosemary-lavender oil, along with rosemary castor oil in comparison to coconut oil, in promoting hair growth and improving scalp health. Despite their widespread traditional use, limited clinical evidence exists on the combined impact of these oils on hair physiology.

Therefore, this study aims to assess their effectiveness in improving hair growth rate, hair density, hair thickness, hair length, and hair fall as primary objectives. By conducting a systematic clinical assessment, this study provides scientific insights into the role of these natural oils in supporting hair health and managing alopecia.

## Materials and methods

Ethical conduct of the study

The research was conducted in accordance with the New Drugs and Clinical Trials Rules, 2019, the International Council for Harmonisation of Technical Requirements for Pharmaceuticals for Human Use (ICH) E6 (R2) Good Clinical Practice guidelines, the Declaration of Helsinki (Brazil, October 2013), and the Indian Council of Medical Research (ICMR) National Ethical Guidelines for Biomedical and Health Research Involving Human Participants, 2017. Prior to study enrolment, all participants provided written informed consent, ensuring they were fully informed about the study's objectives, procedures, confidentiality measures, and their voluntary participation.

Furthermore, the clinical study was officially registered with the Clinical Trial Registry of India (CTRI) under the registration number CTRI/2024/07/070860. Ethical approval for the study protocol was granted by the ACEAS Independent Ethics Committee on July 06, 2024, prior to study initiation. This comprehensive ethical framework was implemented to safeguard the rights, safety, and well-being of all participants, ensuring strict adherence to national and international ethical standards.

Study design

This study was designed as a prospective, interventional, double-blind, randomized, three-arm, placebo-controlled, parallel-group clinical trial to evaluate the safety, efficacy, and in-use tolerability of the rosemary-lavender oil, rosemary-castor oil, and coconut oil in individuals aged 19 to 54 years. The trial was conducted at NovoBliss Research Private Limited, Ahmedabad, India, a contract research organization (CRO) specializing in clinical investigations.

The study included a 90-day treatment period, during which participants were randomly assigned to one of three study groups in a double-blind manner to ensure unbiased assessment. Subject recruitment commenced on August 30, 2024, with the first subject's initial visit, and the study concluded on December 27, 2024, following the last subject's final visit. This structured approach ensured a rigorous evaluation of the investigational products while maintaining adherence to ethical and scientific research standards. 

The primary objective of this study was to evaluate the efficacy of a rosemary-lavender, castor oil, in comparison with the coconut oil that served as a placebo, for controlling hair growth rate, hair density, hair thickness, hair length, and hair fall over a 90-day treatment period. The secondary objective was to assess their impact on hair root strength, scalp condition, anagen-to-telogen (A:T) ratio, and the general appearance of the hair and scalp, providing a comprehensive analysis of their role in promoting overall hair and scalp health.

The study enrolled healthy males and non-pregnant, non-lactating females aged 18 to 55 years with complaints of thinning hair and experiencing a hair fall count of 40-50 (females) or 25-30 (males). Females of childbearing potential required a self-reported negative pregnancy test and adhere to a reliable birth control method. Participants were in general good health, had been on a stable dose of hormonal contraception or therapy for at least six weeks, and committed to avoiding medicated hair products, Minoxidil, or hair dye during the study. All participants provided written informed consent, complied with study procedures, and exclusively used the test product for the study duration.

**Figure 1:**
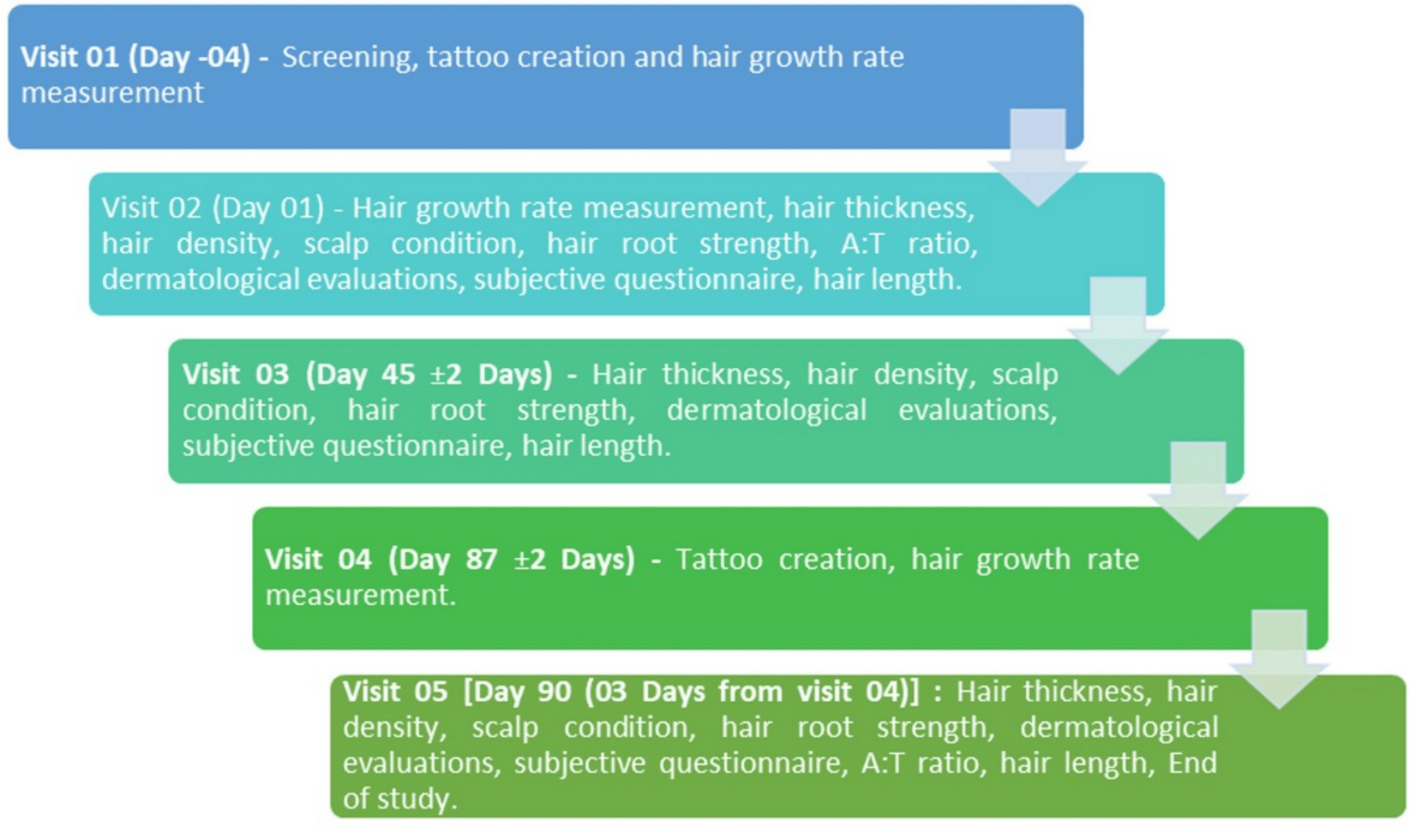
Study visits and procedures

Individuals with scalp conditions (other than hair loss), recent hair treatments, chronic illnesses affecting skin, or patch test irritation were excluded. The study also excluded those using hair loss treatments, salon services, or planning scalp shaving, as well as pregnant/breastfeeding women, allergy-prone individuals, substance users, and those on chronic steroids. Participation in similar trials or hair growth treatments within four weeks prior was not permitted (Figure 1).

The study evaluated three different hair oils: Soulflower Rosemary Lavender Healthy Hair Oil (rosemary lavender hair oil), a blend of Soulflower Rosemary Essential Oil and Soulflower Cold-Pressed Castor Oil (rosemary with castor oil), and Coconut Hair Oil (coconut oil), each with specific formulations and application methods.

Soulflower Rosemary Lavender Healthy Hair Oil, Rosmagain™, marketed by Soulflower Pvt Ltd, contained the Rosmagain complex, a blend of castor oil, sesame oil, coconut oil, rosemary essential oil, olive oil, amla oil, lavender oil, jojoba oil, juniper oil, and vitamin E. Participants applied 15 mL of the oil directly to the scalp, massaging for 5-10 minutes to enhance blood circulation. The oil was left on for at least four hours or overnight before rinsing with a mild shampoo and warm water. 

All products were administered topically, three times a week, following the same application protocol to ensure consistency in usage across study participants.

The CASLite Nova Hair Analysis System (Catseye Systems & Solutions Pvt Ltd, Navi Mumbai, India) is a patented, advanced software-based technology designed for precise measurement and analysis of hair growth rate, hair thickness, hair density, and scalp condition. The study employed standardized and validated methodologies to assess hair growth rate, hair length, anagen-to-telogen ratio, hair density, and hair thickness, ensuring an accurate evaluation of the test treatments. Assessments were conducted four days before day 1, followed by evaluations on day 1, day 45, day 87, and day 90, allowing for a detailed analysis of hair parameters [24,25].

A certified MD dermatologist conducted dermatological evaluations to assess the safety and efficacy of the test treatment on the scalp and hair. These assessments involved both visual and tactile examination techniques to ensure a thorough analysis. Key parameters included scalp health, signs of irritation or adverse reactions (such as redness, itching, or flaking), and overall hair condition, including texture, shine, and strength. Additionally, the severity of graying was evaluated using a standardized scoring scale by an expert dermatologist. 

Sixty-second hair combing method

Hair shedding was assessed using the 60-second hair combing technique. Participants were instructed to flip their hair forward and comb it for 60 seconds over a contrasting-colored sheet using a designated comb. Combing was performed from the back to the front of the scalp. Shed hairs collected from the comb and sheet were counted by trained staff and stored in pre-labelled zip-lock bags. Hairs were categorized and counted based on the presence or absence of a hair bulb (i.e., with bulb and without bulb) and stored separately with appropriate labelling.

Hair pull test - hair strength

The pull test was performed to evaluate the epilability of scalp hair and detect active hair shedding. Approximately 20-60 hairs were grasped close to the scalp between the thumb, index, and middle fingers and gently but firmly pulled along the hair shaft. The number of hairs released was counted. A negative test indicated zero to three hairs shed, slightly positive indicated four to six hairs, and clearly positive indicated more than six hairs (>10% of pulled hairs), suggesting active telogen hair loss.

Hair pluck test - anagen:telogen ratio

The trichogram was performed to evaluate the hair growth cycle by determining the proportion of hairs in the anagen and telogen phases based on morphological root characteristics. Hairs were plucked from two standardized scalp regions - an affected area (e.g., temporal or vertex in androgenetic alopecia) and a non-affected reference area (e.g., occipital region)-using rubber-tipped surgical forceps. Approximately 50-100 hairs were extracted with a swift, firm pull directed away from the scalp. Due to the transient discomfort caused by the procedure, subjects were informed and prepared in advance.

Plucked hairs were immediately mounted root-side into a solution (xylene, DPX, or equivalent) on glass slides, covered with a cover slip, and left to dry for 24 hours. Hair roots were then analyzed under a light microscope at 40× magnification to classify and quantify the percentage of anagen, telogen, catagen, and dystrophic hairs.

Assessment of the general appearance of hair and scalp

The overall appearance of hair, including parameters such as hair volume, density, plasticity, shine, smoothness, dryness, and scalp characteristics, including itchiness, dryness, redness, roughness, and scaliness, was evaluated by the dermatologist and the dermatologist-trained evaluator using a standardized five-point scoring scale [26]. 

Subject response index

Subject-reported outcomes were evaluated through a structured questionnaire designed to assess perceived changes across multiple hair parameters. Participants were asked to rate the extent of change in the following attributes: scalp irritation, hair texture, hair thickness, hair density, hair fall, hair root strength, hair length, and overall satisfaction. Each parameter was rated based on the degree of change (moderate or significant), allowing for a subjective evaluation of product performance and user satisfaction over the 90-day application period. 

Randomization and blinding

This study employed a 1:1:1 randomization ratio to allocate participants into three treatment groups: rosemary lavender hair oil, rosemary with castor oil, and coconut oil. The randomization sequence was generated using R Software (version 4.3.1; R Foundation, Vienna, Austria) by an independent biostatistician to ensure unbiased group allocation. Double blinding was maintained by ensuring the study staff who were involved in product dispensing and distribution were not involved in any other study-related activities, and patients were unaware of the test products given to them.

Statistical analysis

Descriptive statistics were used to summarize continuous variables, including mean, standard deviation (SD), median, minimum, and maximum values. Categorical variables were expressed as frequencies and percentages, with graphical representations where applicable. Statistical analysis was performed using SPSS version 29.0.1.0 (IBM Inc., Armonk, New York) and Excel 2019 (Microsoft, Redmond, Washington), with a 5% level of significance. Data from withdrawn participants were excluded from the analysis.

Data were reviewed for accuracy and completeness before analysis. Frequency analyses and cross-tabulations ensured consistency. Missing data were either imputed or excluded from the analysis based on their nature and extent. Paired t-tests were used for statistical evaluation, with p-values and confidence intervals reported for precision and reliability.

Sample size determination

An appropriate sample size was determined to ensure the validity of the study results. Based on statistical calculations, assuming a common standard deviation (SD) of 1.2 at the end of treatment, a sample size of 30 participants per group was deemed sufficient to detect a mean difference of 0.88 between treatments, with 80% power at a 5% significance level (two-sided test). The estimation was based on key parameters, including a significance level (α) of 0.05, power (1-β) of 0.80, a mean difference (Δ) of 0.88, and a two-sample t-test approach. Considering the literature review and statistical analysis, a total of 30 subjects per group were enrolled in the study.

## Results

Demographics and other baseline characteristics

**Figure 2:**
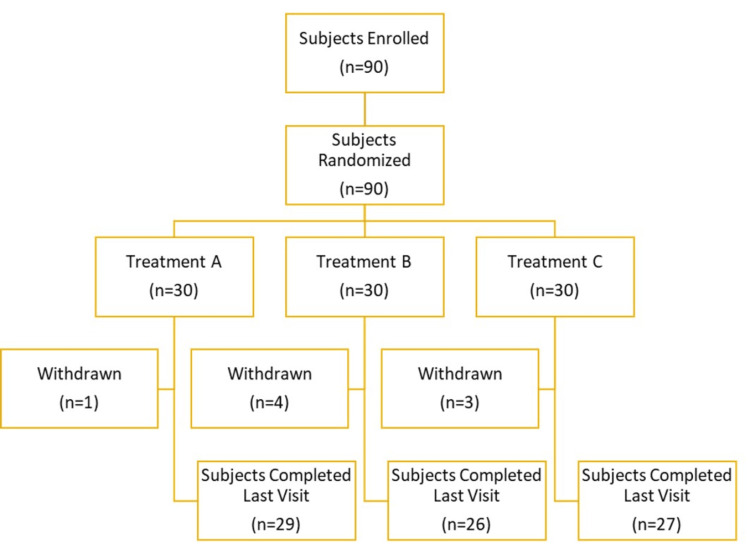
Subject disposition flowchart

The study comprised a total of 90 participants, including 54 females and 36 males. The mean age of the subjects was 38.89 years, weight was 61.30 kg and height was 157.81 cm. Table 1 provides a comprehensive breakdown of demographic characteristics such as gender, age, weight, and height, distributed across different treatment groups (Figure 2).

**Table 1:** Subject demographics and baseline characteristics

Parameter	Statistics	Treatment A (N=29)	Treatment B (N=26)	Treatment C (N=27)	Overall (N=82)
Gender	Male	11 (37.93%)	8 (30.77%)	13 (48.15%)	32 (39.02%)
M / F / TG	Female	18 (62.07%)	18 (69.23%)	14 (51.85%)	50 (60.98%)
Predominant race	Asian	29 (100.00%)	26 (100.00%)	27 (100.00%)	82 (100%)
Medical/ ConMed history (Yes/No)	No	29 (100.00%)	26 (100.00%)	27 (100.00%)	82 (100%)
General well being	Normal	29 (100.00%)	26 (100.00%)	27 (100.00%)	82 (100%)
Age (years)	Mean (SD)	40.24 (9.81)	37.42(7.56)	38.67 (7.75)	38.83 (8.46)
	Median	41.00	40.00	38.00	40.00
	Min, Max	19.00, 53.00	20.00, 51.00	24.00, 54.00	19.00, 54.00
Weight (kg)	Mean (SD)	59.13 (10.77)	61.13 (11.40)	63.03 (8.66)	61.05 (10.34)
	Median	59.12	61.85	62.14	61.44
	Min, Max	36.58, 78.84	45.24, 86.25	44.65, 81.00	36.58, 86.25
Height (cm)	Mean (SD)	157.18 (9.75)	156.56(9.11)	159.88 (10.21)	157.87 (9.7)
	Median	157.00	154.50	159.00	157.00
	Min, Max	139.00, 179.00	142.00, 186.00	140.00, 183.44	139.00, 186.00

Eight subjects were withdrawn from the study due to being lost to follow-up during subsequent evaluation visits. Consequently, these subjects were not included in the statistical analysis to maintain the integrity of the per-protocol analysis (Table 1).

Primary efficacy endpoints

Hair Growth Rate

**Figure 3:**
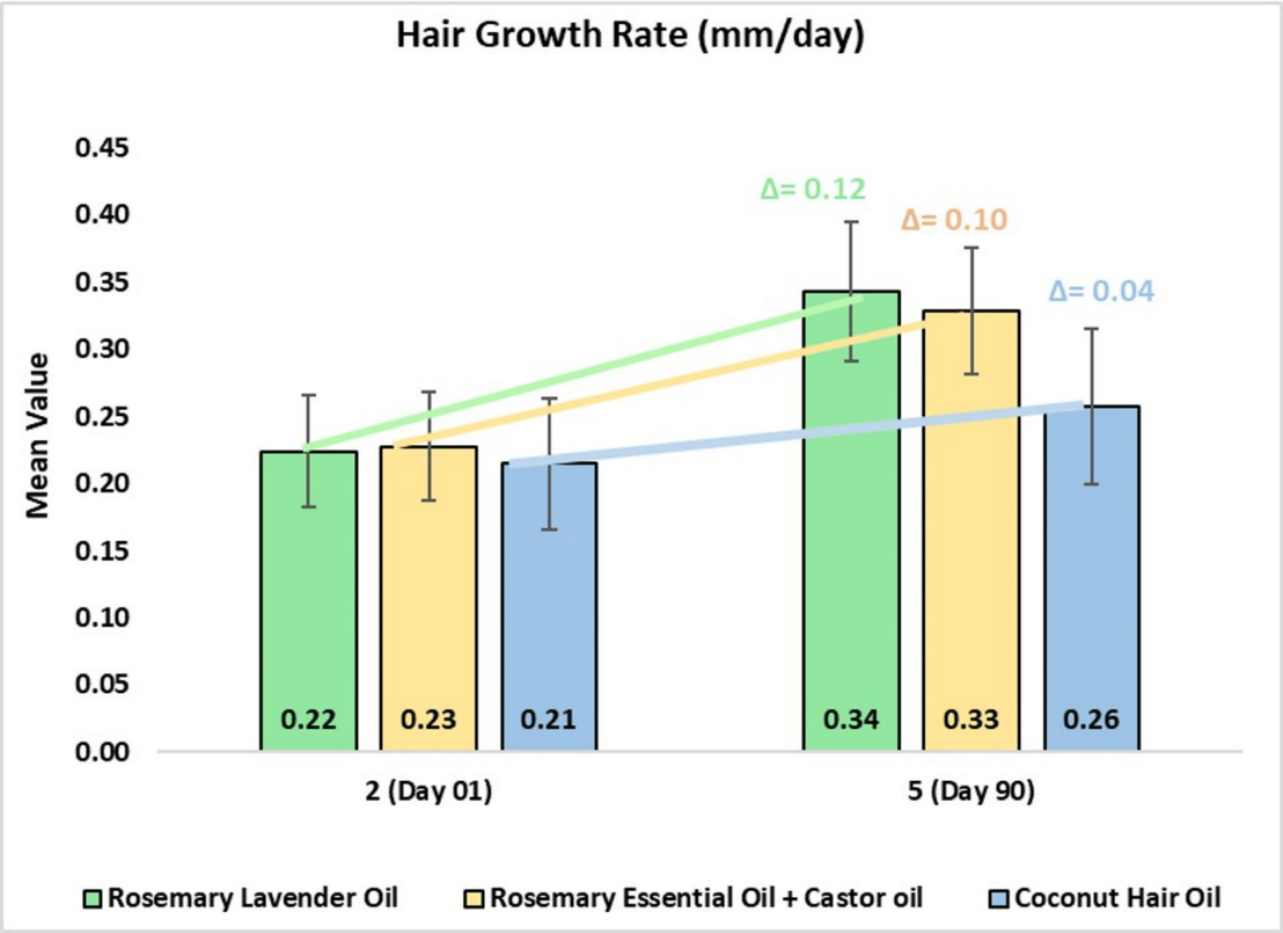
Change in hair growth rate (mm/day) assessed by CASLite Nova

In the rosemary lavender hair oil group, the mean hair growth rate was 0.22 ± 0.04 mm/day on day one and 0.34 ± 0.05 mm/day, or 57.73% change from baseline (CFB), on day 90 (p<0.0001). In the rosemary with castor oil group, the mean hair growth rate was 0.23 ± 0.04 mm/day on day one and 0.33 ± 0.05 mm/day, or 47.59% CFB, on day 90 (p<0.0001). In the coconut oil group, the mean hair growth rate was 0.21 ± 0.25 mm/day on day one and 0.26 ± 0.06 mm/day, or 21.11% CFB, on day 90 (p<0.0001). On comparing rosemary lavender oil to rosemary with castor oil, 1.18-fold improvement in hair growth was observed, and to the coconut oil, 2.80-fold improvement was observed. Further comparing rosemary with castor oil to coconut oil, a 2.37-fold improvement was observed (Figure 3).

Hair Thickness 

**Figure 4:**
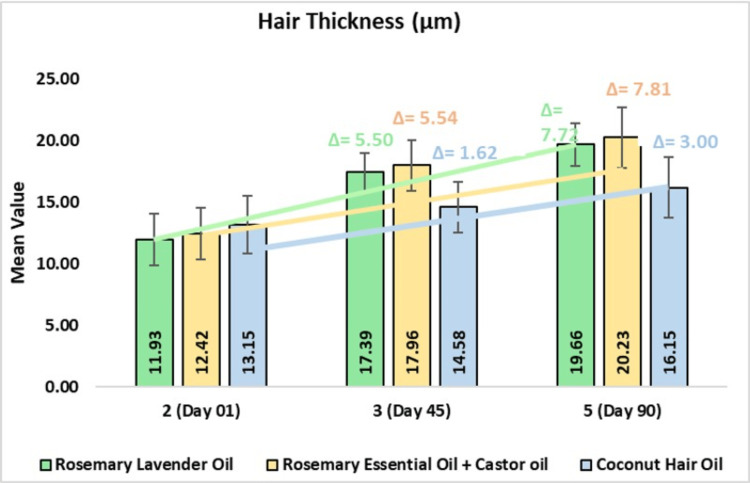
Change in hair thickness (μm) assessed by CASLite Nova

In the rosemary lavender hair oil, the mean hair thickness was 17.39 ± 1.57 µm on day 45 or 48.90% change from baseline (CFB) (p<0.0001) and 19.66 ± 1.76 µm or 68.70% CFB on day 90 (p<0.0001). In the rosemary with castor hair oil group, the mean hair thickness was 17.96 ± 1.59 µm on day 45 or 47.41% CFB (p<0.0001) and 20.23 ± 7.81 µm or 66.07% CFB on day 90 (p<0.0001). In the coconut hair oil group, the mean hair thickness was 14.58 ± 2.08 µm on day 45 or 13.41% CFB (p<0.0001) and 16.15 ± 2.46 µm or 24.34% CFB on day 90 (p<0.0001). On comparing rosemary lavender oil to coconut oil, a 2.57-fold improvement was observed. Further, on comparing rosemary with castor oil to coconut oil, a 2.60-fold improvement was observed (Figure 4).

Hair Density

**Figure 5:**
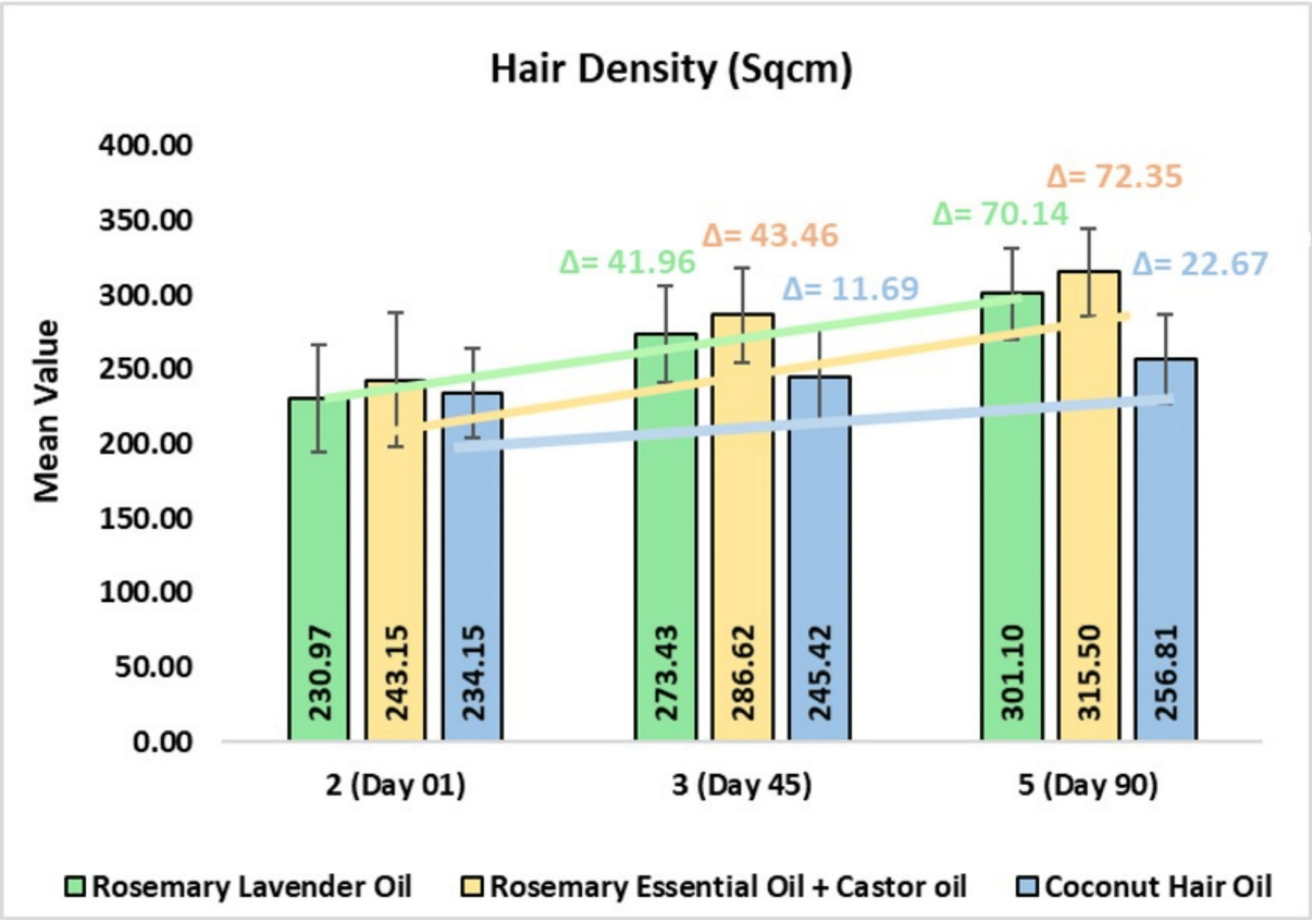
Change in hair density (sqcm) assessed by CASLite Nova

In the rosemary lavender hair oil group, the mean hair density was 273.43 ± 32.52 sqcm on day 45 or 19.18% change from baseline (CFB) (p<0.0001) and 301.10 ± 30.92 sqcm or 32.21% CFB on day 90 (p<0.0001). In the rosemary with castor hair oil group, the mean hair density was 286.62 ± 32.11 sqcm on day 45 or 19.74% CFB (p<0.0001) and 315.50 ± 29.65 sqcm or 32.15% CFB on day 90 (p<0.0001). In the coconut hair oil group, the mean hair density was 245.42 ± 31.51 sqcm on day 45 or 5.13% CFB (p<0.0001) and 256.81 ± 30.03 sqcm or 9.97% CFB on day 90 (p<0.0001). On comparing rosemary lavender oil to coconut oil, a 3.09-fold improvement was observed. Further, on comparing rosemary with castor oil to coconut oil, a 3.19-fold improvement was observed (Figure 5).

Hair Length

**Figure 6:**
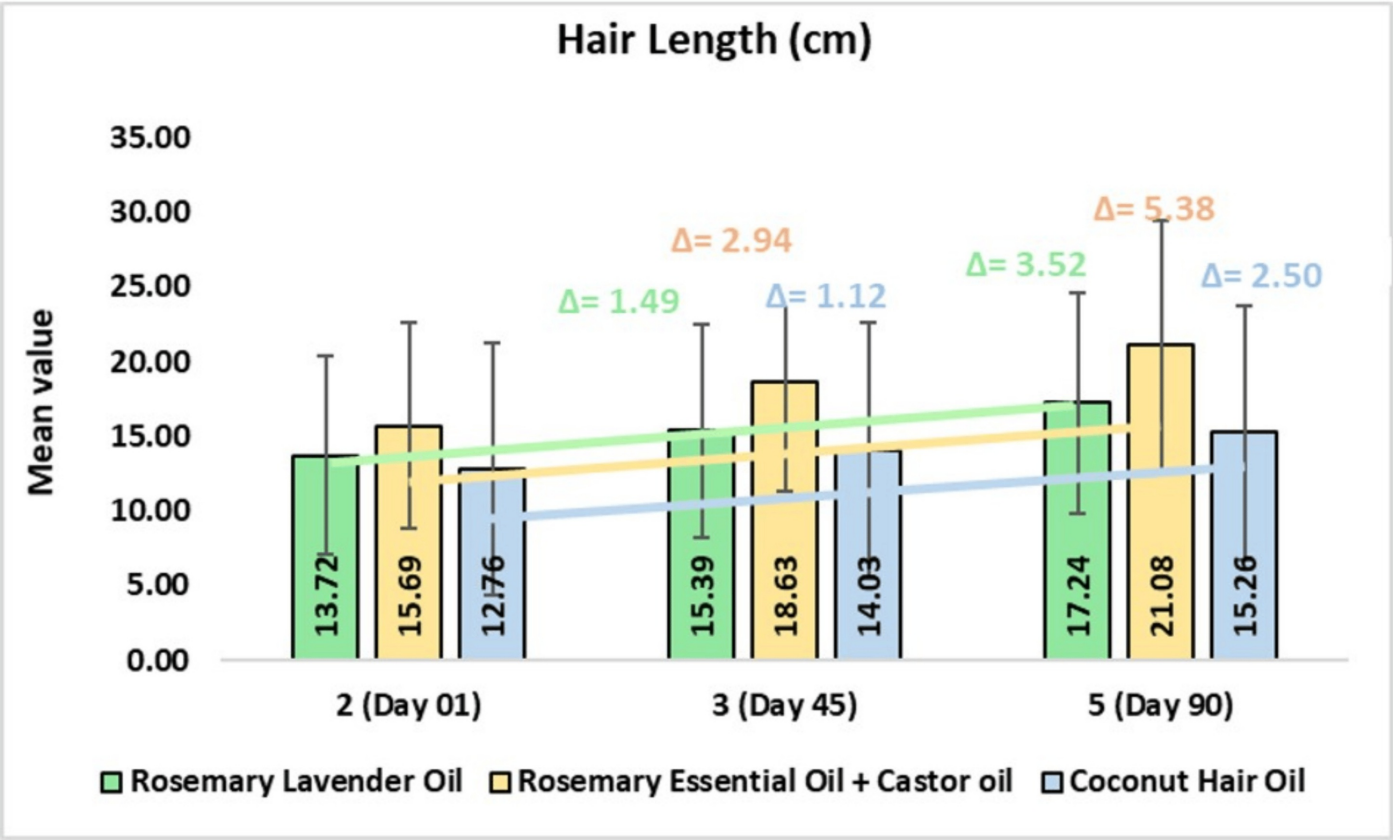
Change in hair length (cm) assessed by CASLite Nova

In the rosemary lavender hair oil group, the mean hair length was 15.39 ± 7.14 cm on day 45 or 11.53% change from baseline (CFB) (p<0.0001) and 17.24 ± 7.39 cm or 28.78% CFB on day 90 (p<0.0001). In the rosemary with castor hair oil group, the mean hair length was 18.63 ± 7.30 cm on day 45 or 21.00% CFB (p<0.0001) and 21.08 ± 8.35 cm or 37.67% CFB on day 90 (p<0.0001). In the coconut hair oil group, the mean hair length was 14.03 ± 8.62 cm on day 45 or 12.03% CFB (p<0.0001) and 15.26 ± 8.53 cm or 26.94% CFB on day 90 (p< 0.0001). On comparing rosemary lavender oil to coconut oil, a 1.41-fold improvement was observed. Further, on comparing rosemary with castor oil to coconut oil, a 2.16-fold improvement was observed (Figure 6).

60-Second Hair Comb Test: Number of Hairs without Hair Bulb

In the rosemary lavender hair oil group, the mean hair without bulb was 22.32 ± 8.98 on day 45 or 17.88% change from baseline (CFB) (p<0.001) and 16.52 ± 7.41 or 40.11% CFB on day 90 (p<0.0001). In the rosemary with castor hair oil group, the mean hair without bulb was 19.38 ± 8.49 on day 45 or 16.23% CFB (p<0.01) and 13.42 ± 6.34 or 42.03% CFB on day 90 (p<0.0001). In the coconut hair oil group, the mean hair without bulb was 22.85 ± 13.40 on day 45 or 15.70% CFB (p<0.01) and 17.44 ± 10.44 or 36.84% CFB on day 90 (p<0.0001). On comparing rosemary with castor oil and coconut oil, a 0.98-fold improvement was observed.

60-Second Hair Comb Test: Number of Hairs with Hair Bulb

In the rosemary lavender hair oil group, the mean hair with bulb was 23.64 ± 9.88 on day 45 or 19.03% change from baseline (CFB) (p<0.001) and 16.69 ± 7.78 or 42.60% CFB on day 90 (p<0.0001). In the rosemary with castor hair oil group, the mean hair with bulb was 24.23 ± 10.45 on day 45 or 26.07% CFB (p<0.001) and 17.27 ± 8.03 or 47.65% CFB on day 90 (p<0.0001). In the coconut hair oil group, the mean hair with bulb was 23.35 ± 16.12 on day 45 or 20.56% CFB (p<0.05) and 18.89 ± 14.51 or 39.64% CFB on day 90 (p<0.0001). On comparing rosemary with castor oil and coconut oil, a 0.98-fold improvement was observed. On comparing rosemary lavender oil to rosemary with castor oil, a 0.84-fold improvement in hair count was observed. Further, on comparing rosemary with castor oil and coconut oil, a 1.47-fold improvement was observed.

60-Second Hair Comb Test: Total Number of Hairs

**Figure 7:**
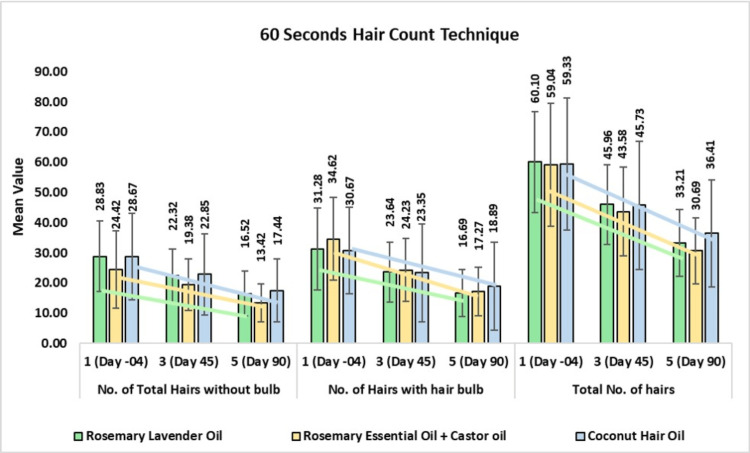
Change in the numbers of hairs without bulb (left) and with bulb (center) and total number of hair fall (right) assessed by the 60-Second Hair Comb Method

In the rosemary lavender hair oil group, the mean total hairs was 45.96 ± 13.22 on day 45 or 23.66% change from baseline (CFB) (p<0.001) and 33.21 ± 11.12 or 44.70% CFB on day 90 (p<0.0001). In the rosemary with castor hair oil group, the mean total hairs was 43.58 ± 14.71 on day 45 or 24.52% CFB (p<0.0001) and 30.69 ± 10.94 or 47.05% CFB on day 90 (p<0.0001). In the coconut hair oil group, the mean hair count was 45.73 ± 21.25 on day 45 or 21.87% CFB (p<0.0001) and 36.41 ± 17.78 or 15.83% CFB on day 90 (p<0.0001). On comparing rosemary with castor oil and coconut oil, a 0.98-fold improvement was observed. On comparing rosemary lavender oil to rosemary with castor oil, a 0.95-fold improvement in hair count was observed (Figure 7).

Secondary efficacy endpoints

Hair Root Strength

On day one before usage of the rosemary lavender oil, 22 (75.86%) subjects had poor and seven (24.14%) subjects had average hair strength. On day 45, seven (25%) subjects had poor, 20 (71.43%) subjects had average, and one (3.57%) subject had good hair root strength. On day 90, nine (31.03%) subjects had average and 20 (68.97%) subjects had good hair strength. 

On day one before usage of the rosemary with castor oil, 15 (57.69%) subjects had poor and 11 (42.31%) subjects had average hair strength. On day 45, seven (26.92%) subjects had poor and 19 (73.08%) subjects had average hair root strength. On day 90, eight (30.77%) subjects had average and 18 (69.23%) subjects had good hair strength.

**Figure 8:**
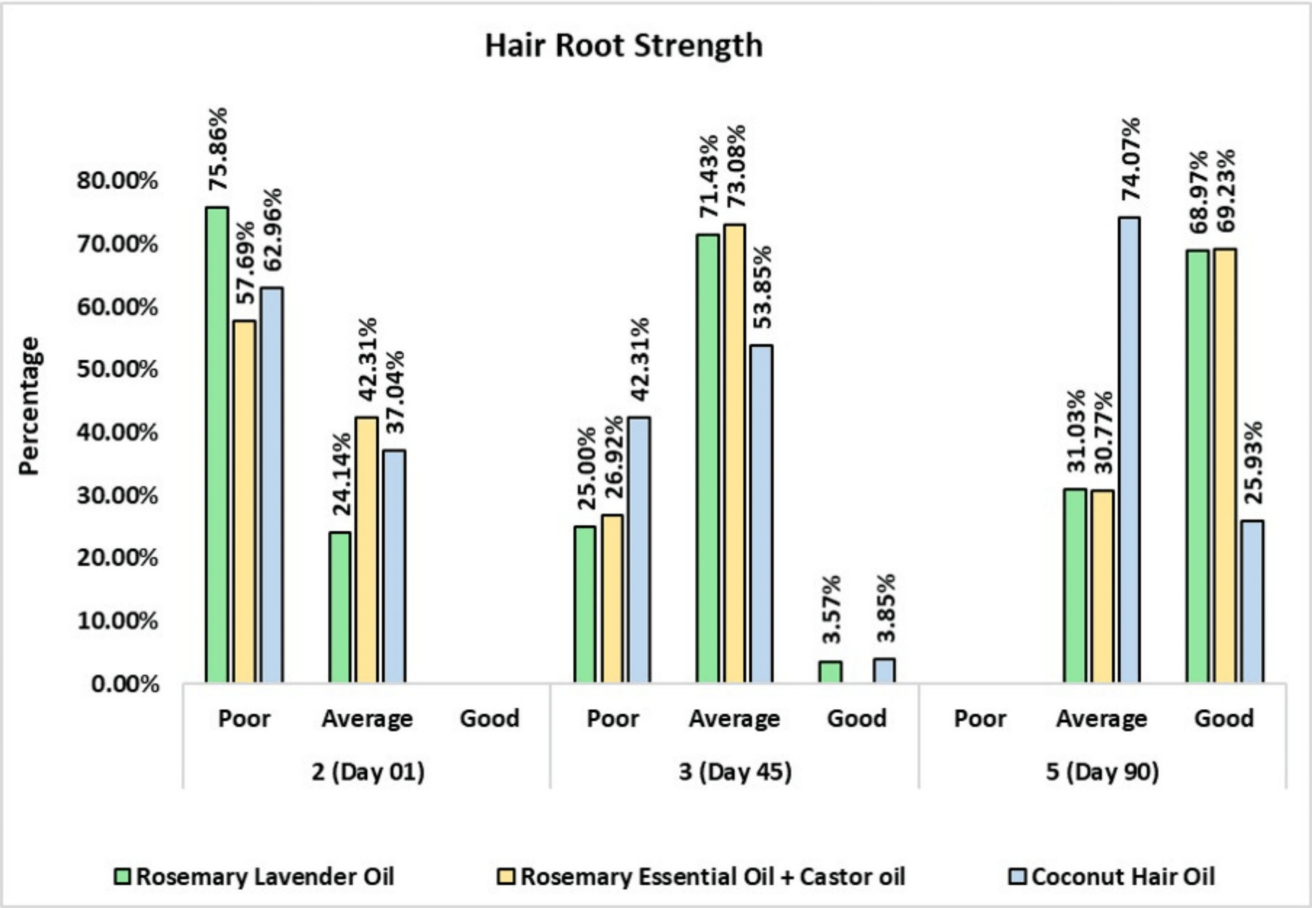
Change in hair root strength

On day one before usage of the coconut oil, 17 (62.96%) subjects had poor and 10 (37.04%) subjects had average hair strength. On day 45, 11 (42.31%) subjects had poor, 14 (53.85%) subjects had average, and one (3.85%) subject had good hair root strength. On day 90, 20 (74.07%) subjects had average and seven (25.93%) subjects had good hair strength (Figure 8).

Scalp Condition

On day one, before use of rosemary lavender hair oil 20 (68.97%) and nine (31.03%) subjects had dry scalp with some keratin and dry scalp with much keratin, respectively. On day 45, 21 (75.00%) subjects had dry scalp with some keratin, four (14.29%) had dry scalp with much keratin, and three (10.71%) had normal scalp with good condition hair thickness and density. On day 90, 24 (82.76%) and five (17.24%) subjects have dry scalp with some keratin and normal scalp with good condition hair thickness and density, respectively.

On day one, before the use of rosemary with castor hair oil, 17 (65.38%) and nine (34.62%) subjects had dry scalp with some and much keratin, respectively. On day 45, 20 (76.92%), four (15.38%), and two (7.69%) subjects have dry scalp with some keratin, much keratin, and normal scalp with good condition hair thickness and density, respectively. On day 90, 15 (57.69%), three (11.54%), and eight (30.77%) subjects have dry scalp with some keratin, dry scalp with much keratin, and normal scalp with good condition hair thickness and density, respectively.

On day one, before use of coconut hair oil, 21 (77.78%), six (22.22%) subjects had dry scalp with some keratin and much keratin, respectively. On day 45, 21 (80.77%), one (3.85%), and four (15.38%) subjects had dry scalp with some keratin, much keratin, and normal scalp with good condition hair thickness and density, respectively. On day 90, 21 (77.78%), one (3.70%), and five (18.52%) subjects had dry scalp with some keratin, much keratin, and normal scalp with good condition hair thickness and density, respectively.

A:T Ratio

**Figure 9:**
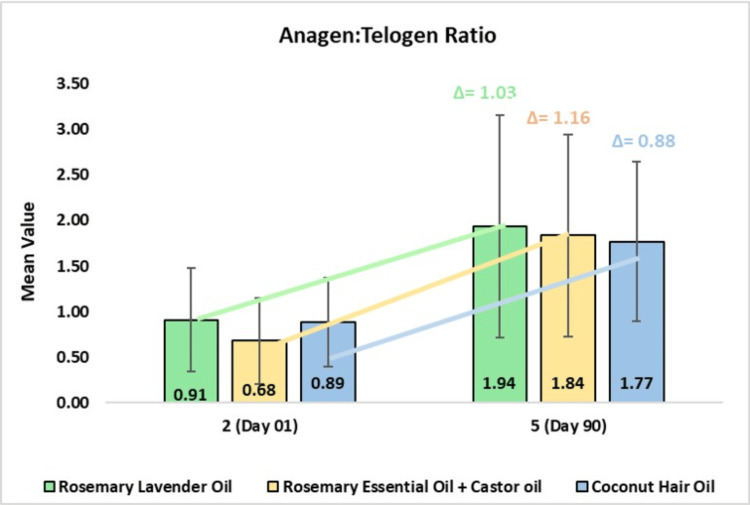
Change in the anagen:telogen ratio

In the rosemary lavender hair oil group, the mean A:T Ratio was 0.91 ± 0.57 on day one and 1.94 ± 1.22 or 184.07% change from baseline (CFB) on day 90 (p<0.001). In the rosemary with castor oil group, the mean A:T Ratio was 0.68 ± 0.47 on day one and 1.84 ± 1.11 or 246.12% CFB on day 90 (p<0.0001). In the coconut oil group, the mean A:T Ratio was 0.89 ± 0.49 on day 01 and 1.77 ± 0.88 or 177.13% CFB on day 90 (p<0.0001). On comparing rosemary lavender oil to rosemary with castor oil, a 0.89-fold improvement in hair growth was observed, and to coconut oil, a 1.17-fold improvement was observed. Further, on comparing rosemary with castor oil to coconut oil, a 1.32-fold improvement was observed (Figure 9).

General appearance of hair

Hair Volume

At baseline, before the use of rosemary lavender hair oil, three (10.34%) subjects had full hair volume, 17 (58.62%) had medium hair volume, and nine (31.03%) had small hair volume. After 45 days of use, three (10.71%) subjects had full hair volume, 21 (75%) had medium hair volume, and four (14.29%) had small hair volume. By day 90, six (20.69%) subjects had full hair volume, and 23 (79.31%) had medium hair volume.

Before the use of rosemary with castor oil, two (7.69%) subjects had full hair volume, 21 (80.77%) had medium hair volume, and three (11.54%) had small hair volume. On day 45, the distribution remained unchanged at two (7.69%), 21 (80.77%), and three (11.54%) for full, medium, and small hair volumes, respectively. By day 90, two (7.69%) subjects had full hair volume, 23 (88.46%) had medium hair volume, and one (3.85%) had small hair volume.

Before the use of coconut oil, one (3.7%) subject had full hair volume, 17 (62.96%) had medium hair volume, and nine (33.33%) had small hair volume. On day 45, one (3.85%) subject had full hair volume, 16 (61.54%) had medium hair volume, and nine (34.62%) had small hair volume. By day 90, one (3.7%) subject had full hair volume, 18 (66.67%) had medium hair volume, and eight (29.63%) had small hair volume.

Hair Shininess

At baseline, before the usage of rosemary lavender hair oil, 28 (96.55%) subjects had poor hair shininess, and one (3.45%) had average shininess. On day 45, 20 (71.43%) subjects had poor shininess, and 8 (28.57%) had average shininess. By day 90, four (13.79%) subjects had poor shininess, while 25 (86.21%) had average shininess.

At baseline, before the usage of rosemary with castor oil, 23 (88.46%) subjects had poor hair shininess, and three (11.54%) had average shininess. On day 45, 15 (57.69%) subjects had poor shininess, while 11 (42.31%) had average shininess. By day 90, three (11.54%) subjects had poor shininess, 21 (80.77%) had average shininess, and two (7.69%) had good shininess.

At baseline, before the usage of coconut oil, 24 (88.89%) subjects had poor hair shininess, and three (11.11%) had average shininess. On day 45, 23 (88.46%) subjects had poor shininess, and three (11.54%) had average shininess. By day 90, 24 (88.89%) subjects had poor shininess, and three (11.11%) had average shininess.

General appearance of ccalp

Skin Itchiness

At baseline, before the usage of rosemary lavender hair oil, eight (27.59%) subjects had no itchiness, 14 (48.28%) had mild itchiness, and seven (24.14%) had moderate itchiness. After 45 days of usage, 26 (92.86%) subjects had no itchiness, while two (7.14%) had mild itchiness. By day 90, 29 (100%) subjects reported no itchiness.

At baseline, before the usage of rosemary with castor oil, four (15.38%) subjects had no itchiness, 15 (57.69%) had mild itchiness, and seven (26.92%) had moderate itchiness. After 45 days, 24 (92.31%) subjects had no itchiness, while two (7.69%) had mild itchiness. By day 90, 26 (100%) subjects reported no itchiness.

At baseline, before the usage of coconut oil, nine (33.33%) subjects had no itchiness, 16 (59.26%) had mild itchiness, and two (7.41%) had moderate itchiness. After 45 days, 14 (53.85%) subjects had no itchiness, while 12 (46.15%) had mild itchiness. By day 90, 26 (96.3%) subjects had no itchiness, while one (3.7%) had mild itchiness.

Skin Roughness

At baseline, before the use of rosemary lavender hair oil, eight (27.59%) subjects had no roughness, 13 (44.83%) had mild roughness, and eight (27.59%) had moderate roughness. After 45 days of usage, 23 (82.14%) subjects had no roughness, while five (17.86%) had mild roughness. By day 90, 29 (100%) subjects reported no roughness.

At baseline, before the usage of rosemary with castor oil, two (7.69%) subjects had no roughness, 18 (69.23%) had mild roughness, and six (23.08%) had moderate roughness. After 45 days, 23 (88.46%) subjects had no roughness, while three (11.54%) had mild roughness. By day 90, 26 (100%) subjects reported no roughness.

At baseline, before the usage of coconut oil, eight (29.63%) subjects had no roughness, 17 (62.96%) had mild roughness, and two (7.41%) had moderate roughness. After 45 days, 14 (53.85%) subjects had no roughness, while 12 (46.15%) had mild roughness. By day 90, 24 (88.89%) subjects had no roughness, while three (11.11%) had mild roughness.

Subject response index

After 90 days, test product A (rosemary lavender hair oil) showed no scalp irritation. Hair texture improved in 44.83% to a moderate extent and 51.72% to a larger extent. Hair thickness and density improved in 51.72% moderately and 48.28% significantly. Hair fall was reduced in 55.17% to a moderate extent and 41.38% to a larger extent. Hair root strength improved in 72.41% moderately and 27.59% significantly. Hair length increased in 48.28% to a moderate extent and 51.72% to a larger extent. Overall, 68.97% were highly satisfied.

For test product B (rosemary with castor oil), 46.15% reported moderate and 53.85% larger improvement in hair texture. Hair thickness improved in 42.31% moderately and 53.85% significantly. Hair fall was reduced in 42.31% to a moderate extent and 57.69% to a larger extent. Hair root strength improved moderately in 57.69% and significantly in 38.46%. Hair length increased in 42.31% moderately and 57.69% significantly. Overall, 76.92% were highly satisfied.

With test product C (coconut oil), 77.78% reported moderate and 11.11% larger improvement in hair texture. Hair thickness improved moderately in 74.07% and significantly in 18.52%. Hair fall reduced moderately in 74.07% and significantly in 14.81%. Hair root strength improved moderately in 62.96% and significantly in 33.33%. Hair length increased in 70.37% moderately and 18.52% significantly. Satisfaction was lower, with 77.78% moderately and 14.81% highly satisfied. 

Photographic assessment

**Figure 10:**
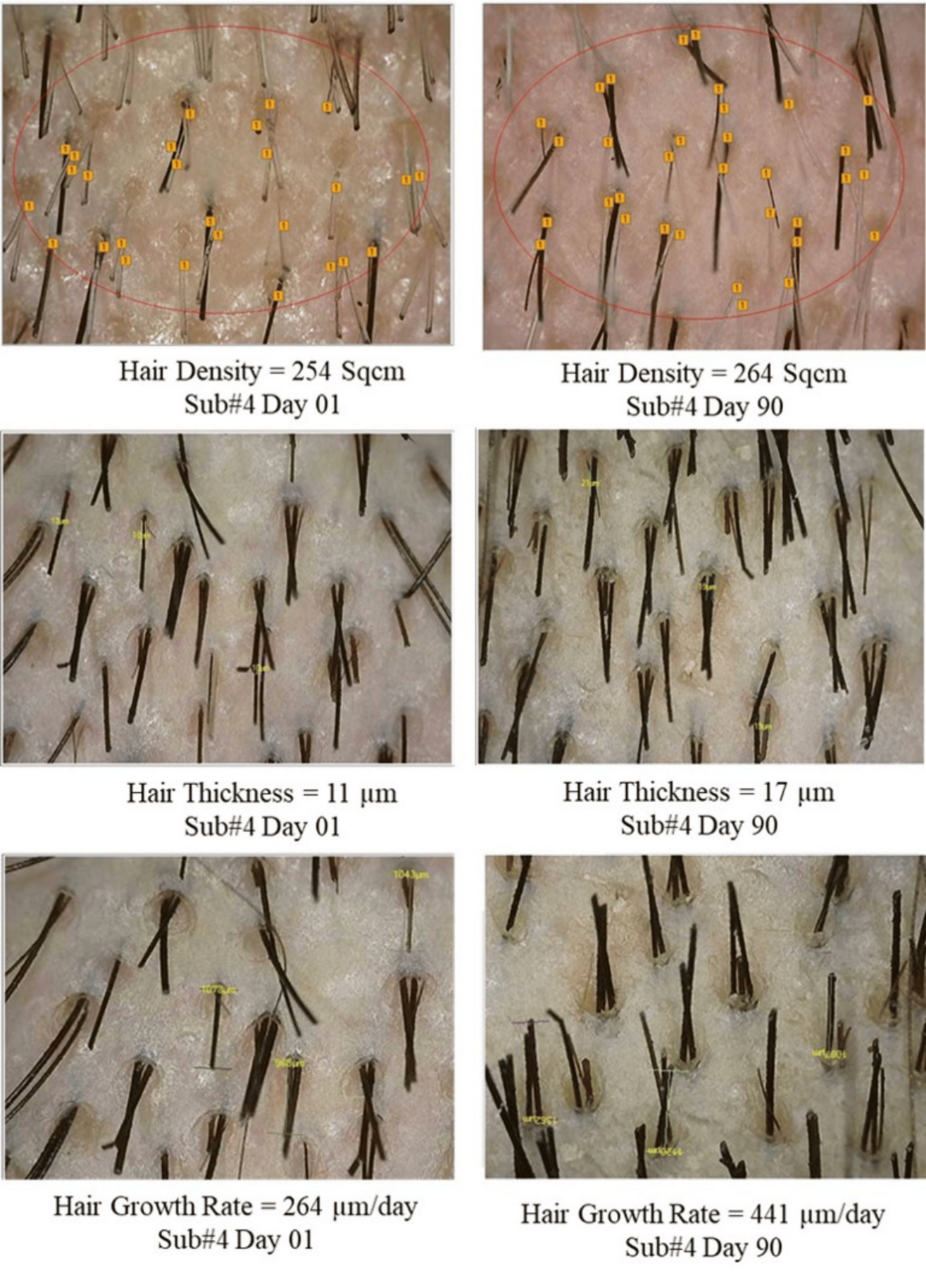
Photographic assessment

In the study, hair growth was evaluated using a detailed photographic assessment. High-resolution images of affected scalp areas were captured under standardized conditions to ensure consistency across all participants (Figure 10).

## Discussion

This study evaluated the efficacy and safety of Soulflower Rosemary Lavender Healthy Hair Oil, Soulflower Rosemary Essential Oil + Cold-Pressed Castor Oil, and Coconut Hair Oil in improving various hair and scalp health parameters. The results demonstrated that the rosemary-based formulations outperformed coconut oil in enhancing hair growth rate, thickness, density, length, root strength, scalp condition, and overall hair appearance, supporting their potential use in hair care.

A significant beneficial effect on hair growth rate was observed with the rosemary-based oils, suggesting their ability to stimulate follicular activity and extend the anagen phase. Previous studies have highlighted rosemary oil's impact on microcirculation and follicular stimulation, which may explain the observed enhancements [22]. Coconut oil, while beneficial for scalp hydration, exhibited comparatively lower efficacy in promoting hair growth.

Both rosemary-based formulations led to a considerable increase in hair thickness and density, indicating their role in improving hair shaft integrity. These findings align with research suggesting that rosemary and castor oils enhance keratin production and strengthen hair strands, leading to thicker, denser hair [27]. Coconut oil, although widely used for scalp nourishment, showed relatively limited impact on hair thickening. Similarly, the improvement in hair length among subjects using rosemary-based formulations further supports their role in prolonging the anagen phase, as reported in prior studies. Castor oil, known for its high ricinoleic acid content, has been suggested to promote hair elongation, complementing the effects of rosemary oil [28]. While coconut oil showed some improvement, its impact was less pronounced compared to the other two formulations [29].

The 60-second hair comb test revealed a significant reduction in hair fall with rosemary-based oils, supporting their effectiveness in reducing excessive shedding and strengthening hair roots. The increased A:T ratio further indicates that these oils help maintain hair in the growth phase for longer durations. The improvement in hair root strength suggests a reinforcing effect on follicular health, likely due to rosemary and castor oil's anti-inflammatory and circulation-enhancing properties.

Dermatological evaluations indicated a notable enhancement in scalp health, with reduced dryness, itchiness, and keratin build-up among subjects using rosemary-based oils. The antimicrobial and anti-inflammatory properties of rosemary and lavender oils may contribute to maintaining a balanced scalp environment, reducing irritation, and promoting optimal hair growth conditions. Subjective assessments further revealed that rosemary-based oils led to improvements in hair volume, shininess, and overall texture, with higher user satisfaction compared to coconut oil. These findings suggest that in addition to promoting hair growth, these formulations enhance the cosmetic appeal of hair, making them valuable for hair care applications.

Several studies have shown rosemary oil to be an effective natural treatment for alopecia, with some findings suggesting efficacy comparable to 2% minoxidil. Research in animal models and human trials supports its ability to stimulate hair follicles, improve scalp circulation, and extend the anagen phase. The results of this study align with these findings, demonstrating significant improvements in hair growth, thickness, density, and scalp health. These outcomes further validate rosemary oil as a promising natural alternative for alopecia management, though further long-term clinical trials are needed.

Studies have shown that rosemary oil formulations can promote hair growth, with a 10% rosemary oil oleogel demonstrating efficacy comparable to 2% minoxidil. However, combining rosemary and cedarwood oils did not enhance results. Further research is needed to optimize essential oil concentrations and assess their long-term safety relative to conventional treatments [30].

Studies have shown that lavender oil-based aromatherapy can be an effective and safe alternative for treating localized alopecia areata. Research comparing aromatherapy treatments with placebo (carrier oils) demonstrated significantly better hair regrowth outcomes with lavender-containing formulations [31]. However, due to limited clinical trials, further research is needed to confirm its long-term efficacy and safety. Given its mild side effect profile, lavender oil may serve as a second-line treatment for alopecia areata, particularly in cases where corticosteroids fail to provide improvement.

This study demonstrates that rosemary-lavender and rosemary-castor oil formulations are effective in enhancing hair growth rate, thickness, density, length, and root strength while improving scalp health and overall hair appearance. Their ability to reduce hair fall and improve follicular health supports their potential as natural alternatives for hair care.

While this clinical study provides valuable insights into the efficacy of rosemary-lavender, rosemary-castor, and coconut oil formulations for hair growth and scalp health, certain limitations should be considered. The study duration was relatively short, and the long-term effects of these treatments remain unknown. Additionally, the sample size was limited, and a larger, more diverse population would strengthen the generalizability of the findings. External factors such as diet, stress levels, and genetic predisposition, which can influence hair growth, were not controlled. Future research with extended follow-up periods, larger cohorts, and controlled external variables is recommended to further validate these results.

## Conclusions

This study provides scientific evidence supporting the efficacy and safety of rosemary-lavender oil, Rosmagain™, and rosemary-castor oil in promoting overall hair and scalp health. Among the tested formulations, rosemary-based oils demonstrated superior performance, showing significant improvements in hair quality and strength. The observed effects suggest that these formulations contribute to maintaining healthy hair growth cycles, making them a promising natural alternative for managing hair concerns.

Additionally, the study highlights the cosmetic and user-perceived benefits of these formulations, with high levels of satisfaction reported among participants. Nonetheless, the findings suggest that rosemary-based oils offer a viable, plant-derived option for individuals seeking non-pharmaceutical solutions for hair and scalp health. 
