# Correction: Rosmagain™ as a Natural Therapeutic for Hair Regrowth and Scalp Health: A Double-Blind, Randomized, Three-Armed, Placebo-Controlled Clinical Trial

**DOI:** 10.7759/cureus.c452

**Published:** 2026-06-17

**Authors:** Maheshvari N Patel, Natasha Tuli, Nayan Patel, Apeksha Merja

**Affiliations:** 1 Clinical Research, NovoBliss Research, Ahmedabad, IND; 2 Pharmacology, Swaminarayan University, Ahmedabad, IND; 3 Research and Development, Soulflower Pvt Ltd, Mumbai, IND; 4 Clinical Research Operations, Novobliss Research Pvt Ltd, Ahmedabad, IND; 5 Clinical Research, Novobliss Research Pvt Ltd, Ahmedabad, IND

Due to a typo, the CTRI number for this study has been corrected to CTRI/2024/07/070860.

